# Peds1 deficiency in zebrafish results in myeloid cell apoptosis and exacerbated inflammation

**DOI:** 10.1038/s41420-024-02141-w

**Published:** 2024-08-29

**Authors:** Ana B. Arroyo, Sylwia D. Tyrkalska, Eva Bastida-Martínez, Antonio J. Monera-Girona, Joaquín Cantón-Sandoval, Martín Bernal-Carrión, Diana García-Moreno, Montserrat Elías-Arnanz, Victoriano Mulero

**Affiliations:** 1 Inmunidad, Inflamación y Cáncer. Departamento de Biología Celular e Histología, Facultad de Biología, Universidad de Murcia, 30100 Murcia, Spain; 2 Instituto Murciano de Investigación Biosanitaria Pascual Parrilla, 30120 Murcia, Spain; 3 Centro de Investigación Biomédica en Red de Enfermedades Raras (CIBERER) Instituto de Salud Carlos III, 28029 Madrid, Spain; 4 Departamento de Genética y Microbiología, Área de Genética (Unidad Asociada al IQFR-CSIC), Facultad de Biología, Universidad de Murcia, 30100 Murcia, Spain

**Keywords:** Cell death and immune response, Immune cell death, Infection, Chronic inflammation

## Abstract

Plasmalogens are glycerophospholipids with a vinyl ether bond that confers unique properties. Recent identification of the gene encoding PEDS1, the desaturase generating the vinyl ether bond, enables evaluation of the role of plasmalogens in health and disease. Here, we report that Peds1-deficient zebrafish larvae display delayed development, increased basal inflammation, normal hematopoietic stem and progenitor cell emergence, and cell-autonomous myeloid cell apoptosis. In a sterile acute inflammation model, Peds1-deficient larvae exhibited impaired inflammation resolution and tissue regeneration, increased interleukin-1β and NF-κB expression, and elevated ROS levels at the wound site. Abnormal immune cell recruitment, neutrophil persistence, and fewer but predominantly pro-inflammatory macrophages were observed. Chronic skin inflammation worsened in Peds1-deficient larvae but was mitigated by exogenous plasmalogen, which also alleviated hyper-susceptibility to bacterial infection, as did pharmacological inhibition of caspase-3 and colony-stimulating factor 3-induced myelopoiesis. Overall, our results highlight an important role for plasmalogens in myeloid cell biology and inflammation.

## Introduction

The function and physicochemical properties of cellular membranes are predominantly determined by the chemical and compositional diversity of lipids. Alterations in membrane lipid homeostasis are related to numerous diseases, but the causality and the underlying molecular mechanisms are often unclear [1]. Glycerophospholipids (GPs) are the major membrane lipids and consist of a glycerol backbone with fatty acids at the *sn*‑1 and *sn*‑2 positions, typically bound via an ester bond. However, a significant subset of GPs, generally known as ether lipids, contain an ether-linked fatty alcohol side chain at the *sn*-1 position [1]. These comprise plasmanyl GPs, with an *sn*-1 alkyl group (1-*O*-alkyl, linked by an ether bond) and plasmenyl GPs, with an *sn*-1 alkenyl group (1-*O*-alk-1′-enyl, with a cis-double bond conjugated with the ether oxygen or vinyl ether bond) [1]. The latter are called plasmalogens and are the most abundant subgroup of ether lipids.

Plasmalogens have a unique distribution across the evolutionary tree, as they are found in strictly anaerobic bacteria, protozoans, and metazoans, where they comprise 5–20% of total GPs content, but are absent in fungi, plants and most aerobic bacteria, except myxobacteria [2, 3]. Plasmalogen composition varies among organisms, organelles, and cell types. In mammals, they are abundant in the brain, heart, kidney, lung, skeletal muscle, and neutrophils, but are scarce in the liver [2, 3]. They are present in nearly all subcellular membranes — plasma membrane, lipid droplets, mitochondria, endoplasmic reticulum, and post-Golgi network — except for peroxisomes, where the early biosynthetic steps occur [2, 3]. Formation of the first intermediate with an ether bond (1-*O*-alkyl-glycerone phosphate) in the peroxisome requires three enzymes: GNPAT (glycerone phosphate *O*-acyltransferase), FAR1/FAR2 (fatty acyl-CoA reductase), and AGPS (alkylglycerone-phosphate synthase). Four more steps in the endoplasmic reticulum lead to the synthesis of plasmanylethanolamine, which is the substrate of PEDS1 (plasmanylethanolamine desaturase 1), an enzyme whose identity as the product of the *TMEM189* gene was only recently discovered [3–6]. PEDS1 generates the vinyl ether bond, producing ethanolamine plasmalogens (PE-PLs), which are converted to the choline form (PC-PLs) in subsequent steps. These are highly enriched in the heart and smooth muscle, while PE-PLs are the most abundant form in the rest of the organs [7].

Besides differing from their ester counterparts, alkyl and alkenyl ether lipids also have different physicochemical properties and, thus, may have different roles in cell physiology. These differences affect membrane packing, curvature and fluidity, as well as interaction with other lipids [8]. Plasmalogens are also proposed to act as molecular scavengers that protect membranes from oxidative damage, since the vinyl ether bond is highly susceptible to cleavage by reactive oxygen species (ROS), yielding products with signaling functions [2]. Until recently, most studies could not discern the specific biological roles of plasmalogens from those of their alkyl ether lipid precursors. However, the discovery of PEDS1 identity has spurred investigation about the exact functions of plasmalogens and the molecular basis of their actions. This orphan enzyme was first identified from delving into the photooxidative stress response of the soil myxobacterium *Myxococcus xanthus*. The study revealed that CarF and its homologs in animals (human, mouse, worm, fly and the two zebrafish paralogs), but not those in plants, encode the long-sought PEDS1 [6]. Soon after, two other studies identified the desaturase for human plasmalogen biosynthesis by completely orthogonal approaches [4, 5].

PEDS1 has been recently involved in cell fitness under hypoxia conditions [9] and in negative regulation of autophagy [10]. A context-dependent role of plasmalogens in ferroptosis has been reported, since some studies find PEDS1 depletion to have no effect on ferroptosis [11, 12], while others suggest a pro-ferroptotic effect [13, 14]. In vivo and in vitro experiments suggest that PEDS1 deficiency reduces progression of gastric and breast cancer [10, 14]. The PEDS1-knockout mouse has several associated phenotypes, such as decreased growth, altered blood parameters and eye abnormalities [15, 16]. However, the molecular basis of these phenotypes remains elusive.

The relevance of ether lipids is highlighted by their involvement in several human pathologies: rare genetic disorders associated with mutations in peroxisomal enzymes involved in ether lipid biosynthesis (Zellweger syndrome and Rhizomelic chondrodysplasia punctata-RCDP) or complex diseases, such as Alzheimer’s and Parkinson’s diseases (AD and PD, respectively), multiple sclerosis, autism, schizophrenia, psychiatric depression, and metabolic and inflammatory disorders, including diabetes mellitus, cancer and various respiratory and cardiac diseases. These data suggest that slight differences in lipid chemical structures are critical for cellular functions [17].

Plasmalogens appear to have a dual role in inflammation. On the one hand, the *sn*-2 position of plasmalogens is mostly occupied by polyunsaturated fatty acids (PUFAs), such as arachidonic acid, which serves as a precursor to produce both pro-inflammatory (i.e. eicosanoids, leukotrienes and thromboxanes) and anti-inflammatory (i.e. lipoxins, resolvins, protectins and maresins) lipid mediators [18]. Intriguingly, the lack of plasmalogens does not necessarily cause a deficit in PUFAs and, thus, they are not essential reservoirs of these lipid mediators [19].On the other hand, plasmalogens have been shown to have anti-inflammatory functions, since plasmalogen replacement therapy (PRT), based on administration of plasmalogens or their precursors to normalize plasmalogens levels in chronic inflammation, which is usually associated to decreased plasmalogens levels, led to promising results in several disease models studies. PRT not only improved phenotypes of patient-derived cells and animal models with peroxisomal disorders and neurodegenerative diseases, but it was also reported to ameliorate symptoms in patients with AD or PD [7, 20]. Despite the observed interrelationship, it is less well understood whether the decrease of plasmalogens is either the cause or the consequence of chronic inflammation-linked diseases.

In the present study, we used the unique advantages of the zebrafish to unequivocally show the relevance of plasmalogens in inflammation. Thus, Peds1 deficiency resulted in neutrophil and macrophage apoptosis and, concomitantly, exacerbated inflammation. Moreover, in a sterile model of acute inflammation, Peds1 deficiency provoked defective inflammation resolution and tissue regeneration, accompanied by neutrophil persistence and predominantly pro-inflammatory macrophages at the injury site. Additionally, Peds1 deficiency also aggravated chronic skin inflammation and caused hyper-susceptibility to bacterial infection.

## Results

### Inhibition of plasmalogen synthesis promotes a proinflammatory stage with increased neutrophil and macrophage apoptosis

To characterize the specific role of plasmalogens in vivo under physiological conditions, single-cell stage eggs were microinjected with specific crRNA/Cas9 complexes (cr*peds1a* and cr*peds1b*) to inhibit the two zebrafish *peds1* paralogs [6] (Supplementary Fig. 1A). High *peds1* knockdown efficiency was achieved with a concomitant reduction of total plasmalogen levels in cr*peds1* larvae (estimated by measuring the dimethylacetal or DMA derivatives; see Methods), which was more pronounced at 5 dpf (Supplementary Fig. 1B). This may be related to maternal transfer of plasmalogens, since we found that plasmalogen content in control larvae was 2-fold higher at 5 dpf than at 3 dpf. Specific DMAs were either decreased (C17:0, C:18:0) or became undetectable (C18:1) in Peds1-deficient larvae relative to control larvae (Supplementary Fig. 1C).

**Fig. 1:**
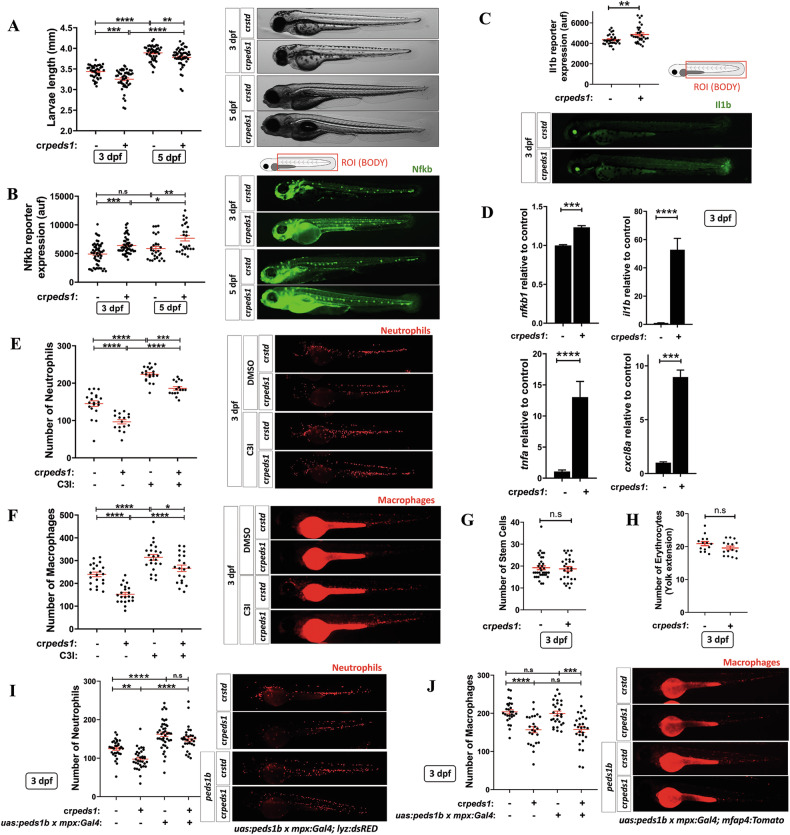
Peds1 deficiency promotes a proinflammatory stage with increased neutrophil and macrophage apoptosis. One-cell stage zebrafish eggs were microinjected with *std* or *peds1* crRNA/Cas9 complexes and the following parameters were evaluated: (**A**) Larval length and representative images (brightfield channel) at 3- and 5 dpf, (**B**) Nfkb fluorescent reporter expression levels and representative images (green channel; *Tg(NFkB-RE:eGFP)*) at 3 and 5 dpf analyzed by fluorescence microscopy, (**C**) Il1b fluorescent reporter expression levels and representative images (green channel; *Tg(il1b:GFP-F)*^*ump3*^) at 3 dpf analyzed by fluorescence microscopy. **D** Transcript levels of the indicated genes analyzed at 3 dpf by RT-qPCR. **E**, **F** Number of neutrophils and macrophages and representative images of microinjected larvae after 48 h of treatment with caspase 3 inhibitor (C3I) or vehicle (DMSO) were quantitated by fluorescence microscopy (red channel; *Tg(lyz:DsRED2)* and *Tg(mfap4.1:Tomato)*, respectively). **G**, **H** Hematopoietic stem and progenitor cells (green channel; *Tg(-6.0itga2b:eGFP)*) and erythrocytes (red channel: *Tg(gata1a:dsRED)*) number were quantitated by fluorescence microscopy. **I**, **J** Number of neutrophils and macrophages and representative images of larvae microinjected to specifically express *peds1* in neutrophils using the neutrophil-specific myeloperoxidase (*mpx*) promoter and analyzed by fluorescence microscopy. Each point represents one larva and the mean ± SEM of each group is also shown. *P-*values were calculated using one-way ANOVA Tukey’s and multiple range or unpaired Student’s *t*-test. n.s, not significant, *p ≤ 0.05, **p ≤ 0.01, ***p ≤ 0.001, ****p ≤ 0.001. auf arbitrary units of fluorescence.

Peds1-deficient larvae were slightly smaller than control larvae at both 3 and 5 dpf (Fig. 1A), but they did not show any obvious developmental defect. Notably, Peds1 deficiency resulted in robust induction of Nfkb and Il1b fluorescent transgene reporters (Fig. 1B, C). In agreement, increased transcript levels of endogenous *nfkb1* and a strong induction of *il1b, tnfa* and *cxcl8a* expression was also observed when plasmalogen synthesis was inhibited (Fig. 1D). Moreover, and surprisingly, the total number of neutrophils and macrophages was lower in cr*peds1* larvae than in their wild-type siblings, and the observed neutropenia and monocytopenia was fully reversed upon pharmacological inhibition of caspase-3 (Fig. 1E, F). However, there were no differences between the two larval groups in the numbers of hematopoietic stem and progenitor cells (HSPCs) (Fig. 1G) or of erythrocytes (Fig. 1H).

We next wondered whether the effect of Peds1 deficiency in neutrophils and macrophages was cell autonomous. The results showed that specific expression of *peds1b* in neutrophils using the neutrophil-specific myeloperoxidase (*mpx*) promoter was able to restore the number of neutrophils (Fig. 1I), but not of macrophages (Fig. 1J), in Peds1-deficient larvae. Collectively, these results suggest a cell autonomous effect of Peds1 in myeloid cells and the non-transferability of plasmalogens between these two cell types.

### Inhibition of plasmalogen synthesis exacerbates chronic skin inflammation

**Fig. 2:**
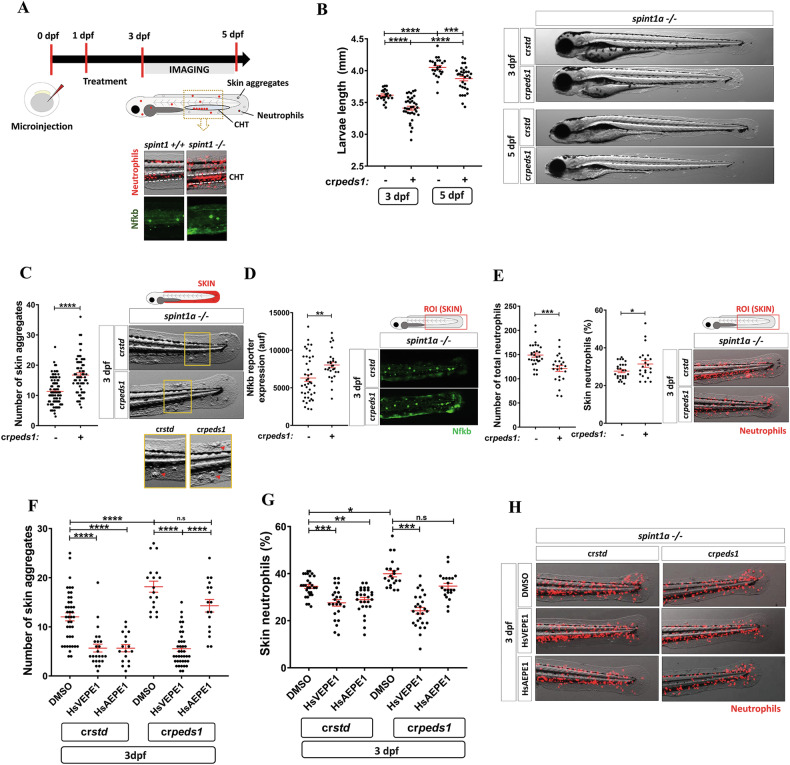
Peds1 deficiency exacerbates chronic skin inflammation. **A** Schematic of the experimental procedure used for chronic skin inflammation assays. Spint1a-deficient one-cell stage eggs were microinjected with *std* or *peds1* crRNA/Cas9 complexes and imaged at 3 and 5 dpf. Treatments of interest were added to dechorionated embryos at 1 dpf by bath immersion and renewed daily. Spint1a-deficient larvae showed chronic skin inflammation with increased skin neutrophil infiltration, Nfkb fluorescent reporter expression levels and keratinocyte aggregates. **B** Analysis and representative images (brightfield channel) of larval length at 3 and 5 dpf. **C** Number of keratinocyte aggregates in the skin (red arrows) and representative images (brightfield channel) at 3 dpf. **D** Analysis and representative images of Nfkb fluorescent reporter expression levels (green channel; *Tg(NFkB-RE:eGFP)*^*sh235*^) at 3 dpf by fluorescence microscopy. **E** Total number and percentage of neutrophils in the skin and representative merge images (brightfield and red channel; *Tg(lyz:DsRED2)*^*nz50*^) quantitated by fluorescence microscopy. **F** Number of skin aggregates and (**G**) percentage of skin neutrophils quantitated in cr*std* and cr*peds*1 *spint1a-/-* larvae treated for 48 h with 20 µM HsVEPE1, 20 µM HsAEPE1 or vehicle (DMSO). **H** Representative merge images (brightfield and red channel; *Tg(lyz:DsRED2)*^*nz50*^) of each treatment analyzed in (**F**) and (**G**) by microscopy fluorescence are shown. Each point represents one larva and the mean ± SEM of each group is also shown. *P-*values were calculated using one-way ANOVA and Tukey’s multiple range or unpaired Student’s *t*-test, as appropriate. n.s, not significant, *p ≤ 0.05, **p ≤ 0.01, ***p ≤ 0.001, ****p ≤ 0.001. auf arbitrary units of fluorescence.

The above results prompted us to study the impact of plasmalogens in chronic inflammation using a Spint1a-deficient line (*spint1a*^*hi2217Tg/hi2217Tg*^) [21], which is characterized by the presence of skin lesions or keratinocyte aggregates, neutrophil mobilization from the caudal hematopoietic tissue (CHT) to inflamed skin, and neutrophilia (Fig. 2A) [22]. We observed again that Peds1-deficient larvae were slightly smaller than their control counterparts (Fig. 2B). In addition, Peds1 deficiency led to increased skin lesions (Fig. 2C) and Nfkb fluorescent reporter expression levels (Fig. 2D) at 3 dpf and, even at 5 dpf, when the skin inflammation was largely resolved in control larvae (Supplementary Figs. 2A, B). Moreover, Peds1-deficient larvae had increased neutrophil infiltration into the skin both at 3 and 5 dpf (Fig. 2E and Supplementary Fig. 2C), even though they had an overall lower number of neutrophils than control larvae (Fig. 2E).

Since inhibition of plasmalogen biosynthesis worsened skin inflammation in Spint1a-deficient larvae, we tested the effect of exogenous supplementation of dechorionated embryos with a commercially available human plasmalogen (HsVEPE1) that has a single double bond at the *sn*‑2 position (see Methods). Importantly, HsVEPE1 alleviated skin inflammation, assayed as the number of keratinocyte aggregates and neutrophil infiltration (Supplementary Fig. 2D). Subsequently, we performed the same experiment but adding HsVEPE1 or its alkyl ether precursor HsAEPE1 to Peds1-deficient and control larvae. Interestingly, both HsVEPE1 and HsAEPE1 decreased skin lesions and neutrophil infiltration in control larvae (Fig. 2F–H). However, in Peds1-deficient larvae, only HsVEPE1 was able to decrease keratinocyte aggregates and neutrophil infiltration (Fig. 2F–H) and to rescue larval length (Supplementary Fig. 2E), likely due to the inability of these larvae to convert HsAEPE1 into HsVEPE1. As the presence of PUFAs in plasmalogens may increase their antioxidant properties, we also tested two plasmalogens enriched in PUFAs, namely HsVEPE2 and HsVEPE3 (with four and six double bonds, respectively, in the acyl chain at *sn*‑2; see Methods). Both plasmalogens ameliorated skin inflammation in Spint1a-deficient larvae, but to levels that resembled those observed with HsVEPE1 (Supplementary Fig. 2F). Overall, these results demonstrate the anti-inflammatory function of plasmalogens, but not of their precursors, in this chronic skin inflammation model.

### Inhibition of plasmalogen synthesis impairs inflammation resolution and tissue regeneration

**Fig. 3:**
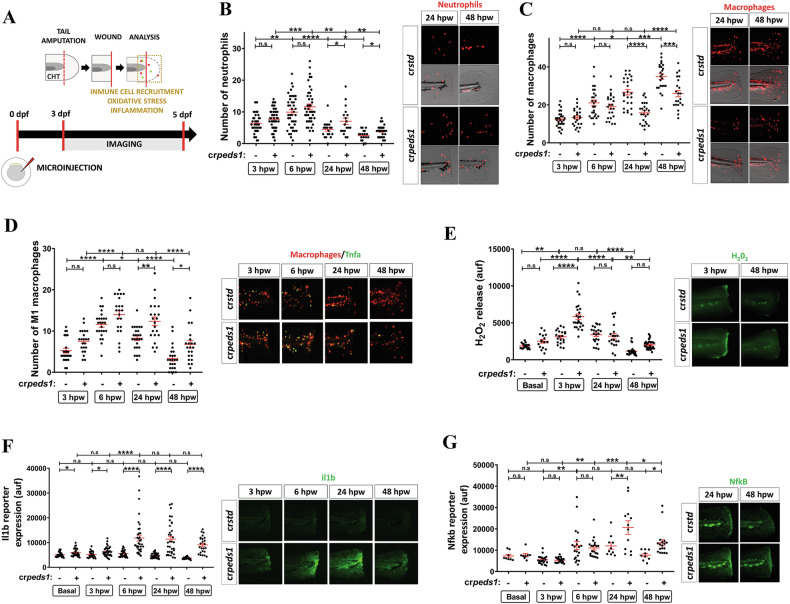
Peds1 deficiency impairs inflammation resolution and causes aberrant immune cells recruitment in a sterile tail injury model. **A** Schematic of the experimental procedure used for tail injury assays. One-cell stage zebrafish eggs were microinjected with *std* or *peds1* crRNA/Cas9 complexes. At 3 dpf larvae were tail amputated and then inflammation and immune cell recruitment at the injury site (dotted lines) were analyzed at 3, 6, 24 and 48 h post-wounding (hpw) by fluorescence microscopy. The wound area is defined as the region between the amputation edge and the end of the caudal hematopoietic tissue (CHT). **B**, **C** Number of neutrophil and macrophages recruited at the injury site and representative merge images (brightfield and red channel; *Tg(lyz:DsRED2)*^*nz50*^ and *Tg(mfap4.1:Tomato)*^*xt12*^, respectively). **D** Number of M1-like macrophages (Tnfa^+^) recruited at the injury site and representative merge images (red channel: *Tg(mfap4.1:Tomato)*^*xt12*^ and green channel: *Tg(tnfa:eGFP-F)*^*ump5*^). **E** Analysis of H_2_O_2_ release at the wound site using the fluorogenic substrate acetyl-pentafluor-obenzene sulphonyl fluorescein. **E**–**G** Analysis and representative images of Il1b (green channel; *Tg(il1b:GFP-F)*^*ump3*^) and Nfkb fluorescent reporter expression levels (green channel; *Tg(NFkB-RE:eGFP)*^*sh235*^) at the wound site. Each point represents one larva and the mean ± SEM of each group is also shown. *P-*values were calculated using one-way ANOVA and Tukey’s multiple range. n.s, not significant, *p ≤ 0.05, **p ≤ 0.01, ***p ≤ 0.001, ****p ≤ 0.001. auf arbitrary units of fluorescence.

The effect of inhibiting plasmalogen synthesis under acute sterile inflammation was also evaluated. For this purpose, Peds1-deficient larvae were tail-cut and neutrophil and macrophage recruitment, oxidative stress and inflammation were analyzed (Fig. 3A). Although recruitment to wound at early times (3- and 6-h post-wounding, hpw) was normal in Peds1-deficient larvae, at 24 and 48 hpw they showed higher neutrophil (Fig. 3B) and lower macrophage (Fig. 3C) recruitment than control larvae. However, the number of proinflammatory macrophages, i.e. M1-like that express a Tnfa reporter [23], was higher in Peds1-deficient larvae at all times evaluated (Fig. 3D). Oxidative stress, as assessed by hydrogen peroxide release, was found to be higher in larvae deficient in Peds1 at 3 hpw (Fig. 3E). In agreement with the above data, Peds1 deficiency caused a dramatic increase in Il1b (Fig. 3F) and Nfkb (Fig. 3G) fluorescent reporter expression levels at 24 and 48 hpw. These results suggest that Peds1-deficient larvae fail to resolve inflammation.

**Fig. 4:**
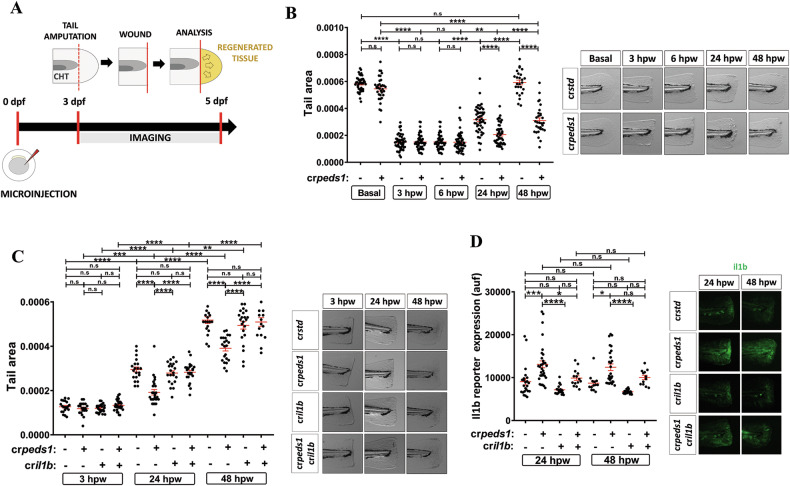
Peds1 deficiency hampers tissue regeneration. **A** Schematic of the experimental procedure used for tail injury and regeneration assays. Three dpf larvae previously microinjected with *std* or *peds1* crRNA/Cas9 complexes alone or in combination with cr*il1b* were tail fin amputated and regeneration was evaluated by measuring regenerated fin areas up to 5 dpf (yellow). **B**, **C** Measurement of regenerated fin areas and representative images (brightfield channel). **D** Analysis and representative images of Il1b fluorescent reporter expression levels (green channel; *Tg(il1b:GFP-F)*^*ump3*^) by microscopy fluorescence. Each point represents one larva and the mean ± SEM of each group is also shown. *P-*values were calculated using one-way ANOVA and Tukey’s multiple range. n.s, not significant, *p ≤ 0.05, **p ≤ 0.01, ***p ≤ 0.001, ****p ≤ 0.001. auf arbitrary units of fluorescence.

Since Peds1 deficiency altered the inflammatory response to provoked injury, its effect on tissue regeneration was next determined (Fig. 4A). Unlike control larvae, which completely regenerated the tail at 48 hpw, Peds1-deficient larvae failed to do so (Fig. 4B). Therefore, we wondered whether this impaired regeneration of Peds1-deficient larvae was due to failure in resolving inflammation or, alternatively, to plasmalogens being required for cell proliferation. As Il1b was dramatically induced in the wound of Peds1-deficient larvae, but it hardly increased in control larvae, we analyzed the effect of knocking down this proinflammatory cytokine (knockout efficiency of about 80%) (Supplementary Fig. 3). While Il1b deficiency had no effect on tail regeneration of control larvae, it fully rescued regeneration and inflammation, assayed as Il1b fluorescent reporter expression levels, in Peds1-deficient larvae (Fig. 4C, D). Therefore, the inability of Peds1-deficient larvae to resolve inflammation appears to be responsible for the impaired tissue regeneration in this acute inflammation model.

### Inhibition of plasmalogen synthesis results in increased susceptibility to infection

**Fig. 5:**
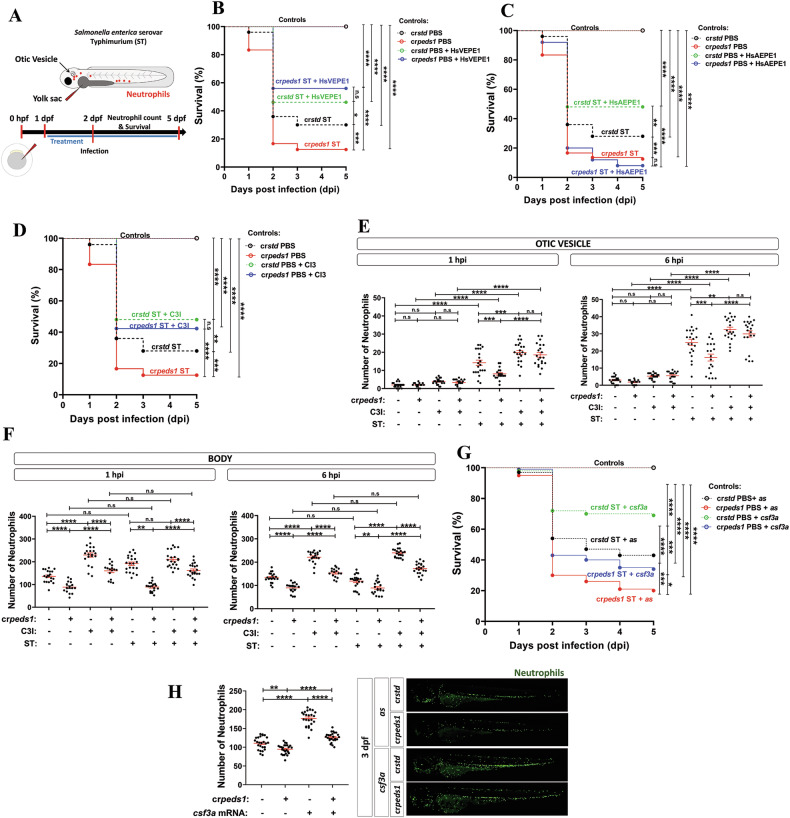
Peds1 deficiency increased susceptibility to bacterial infection. **A** Schematic of the experimental procedure used for infection assays. Single-cell zebrafish embryos microinjected with *std* or *peds1* crRNA/Cas9 complexes were dechorionated and infected at 2 dpf through either the otic vesicle (neutrophil recruitment) or yolk sac (larval survival) with *Salmonella enterica* serovar Thyphimurium (ST). Treatments of interest were added daily from 1 dpf by immersion and the number of surviving larvae was counted daily for the next 5 days. **B** Survival analysis of cr*std* and cr*peds1* larvae infected with ST and treated with 20 µM HsVEPE1 or vehicle (DMSO). **C** Survival analysis of cr*std* and cr*peds1* larvae infected with ST and treated with 20 µM HsAEPE1 or vehicle (DMSO). **D** Survival analysis of cr*std* and cr*peds1* larvae infected with ST and treated with caspase 3 inhibitor (C3I, 50 µM) or vehicle (DMSO). **E**, **F** Number of neutrophil in the otic vesicle and body at 1 and 6 h post-infection in cr*std* and cr*peds1* larvae treated with 50 µM C3I or vehicle (DMSO) counted by fluorescence microscopy (red channel; *Tg(lyz:DsRED2)*^*nz50*^). **G** Survival analysis of cr*std* and cr*peds1* larvae injected in combination with antisense (As) or *csf3a* mRNA and infected with ST. **H** Number of neutrophil in whole body in uninfected cr*std* and cr*peds1* larvae of 3 dpf treated with 50 µM C3I or vehicle (DMSO) counted by fluorescence microscopy (green channel; *(mpx:eGFP)*^*i114*^). **B**–**D** and **G** A log-rank test was used to calculate the statistical differences in the survival of the different experimental groups. **E**, **F** Each point represents one larva and the mean ± SEM of each group is also shown. *P-*values were calculated using one-way ANOVA and Tukey’s multiple range. n.s not significant, *p ≤ 0.05, **p ≤ 0.01, ***p ≤ 0.001, ****p ≤ 0.001.

The effect of inhibiting plasmalogen synthesis on the resistance to infection by the intracellular bacterium *Salmonella enterica* serovar Thyphimurium (ST), upon microinjecting it in the yolk sac, was also evaluated (Fig. 5A). The results showed that Peds1-deficient larvae were more susceptible to ST than their wild type siblings (Fig. 5B). Notably, supplementation with exogenous plasmalogen (HsVEPE1) not only mitigated the higher susceptibility of Peds1-deficient larvae to ST infection, but it also robustly increased the resistance of wild type larvae (Fig. 5B). As expected, supplementation with the plasmalogen precursor (HsAEPE1) increased infection resistance of control larvae but failed to alleviate the higher susceptibility to ST of Peds1-deficient larvae (Fig. 5C). Since neutrophils play a critical role in the resistance of zebrafish larvae to ST [24], we tested the effects of inhibiting their apoptosis in infected Peds1-deficient larvae and found that the caspase-3 inhibitor phenocopied the effects of plasmalogen supplementation in larval survival (Fig. 5D). Furthermore, inhibition of caspase-3 also rescued neutrophil recruitment to the otic vesicle of infected Peds1-deficient larvae (Fig. 5E). Regarding the impact of neutrophil apoptosis inhibition in total neutrophil number of Peds1-deficient larvae, Peds1 deficiency resulted in reduced neutrophil number in both uninfected and infected larvae, and caspase-3 inhibition was able to normalize the neutrophil counts (Fig. 5F). Finally, the relevance of neutropenia in the higher susceptibility of Peds1-deficient larvae to ST was further confirmed by the ability of granulocyte colony-stimulating factor (*csf3a*), which induces neutrophil production, to robustly increase the resistance of wild type larvae and partially rescue the resistance (Fig. 5G) and neutrophil number of Peds1-deficient larvae (Fig. 5H), when *csf3a* was forced to express by mRNA microinjection (see Methods).

### Peds1a/b double knock-out (DKO) line shows developmental delay, neutropenia, monocytopenia and exacerbated inflammation

**Fig. 6:**
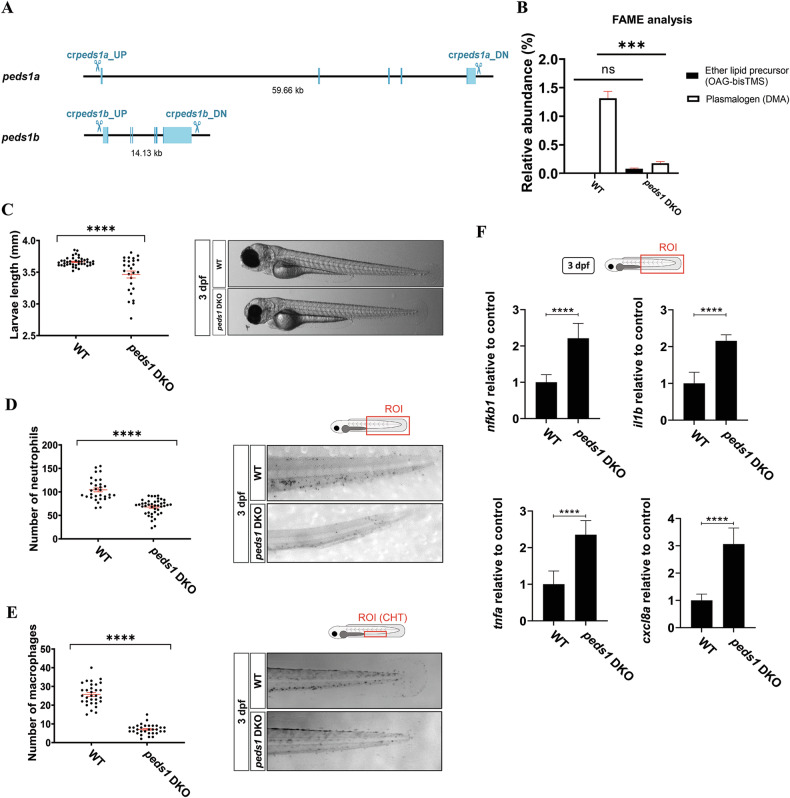
Peds1a/b DKO line shows developmental delay, neutropenia, monocytopenia and exacerbated inflammation. **A** Generation of a DKO line by whole deletion of *peds1a* and *peds1b* genes using CRISPR-Cas9. **B**–**F** Total plasmalogen and precursor quantification (**B**), larval length (**C**), number of neutrophil (**D**) and macrophage (**E**), and transcript levels of *nfkb1*, *il1b*, *tnfa* and *clxcl8a* genes, assayed by RT-qPCR (**F**). Each point represents one larva and the mean ± SEM of each group is also shown. *p ≤ 0.05, **p ≤ 0.01, ***p ≤ 0.001. ROI region of interest, CHT caudal hematopoietic tissue.

To further confirm the relevance of plasmalogens in myeloid cell biology and inflammation, we generated a DKO line by deleting the entire *peds1a* and *peds1b* genes (Fig. 6A). The DKO line showed no obvious morphological alterations and showed normal mendelian ratio. As with the analyses reported above using the knockdown lines, Peds1 deficiency resulted in reduced plasmalogens levels and increased precursors (low levels of plasmalogen remained, likely due their acquisition from the mutant mother, who obtains them from her diet; Fig. 6B), slight developmental delay (Fig. 6C), neutropenia (Fig. 6D), monocytopenia (Fig. 6E), and exacerbated inflammation, assayed as increased transcript levels of *nfkb1*, *il1b*, *tnfa* and *cxcl8a* genes (Fig. 6F), at 3 dpf.

## Discussion

**Fig. 7:**
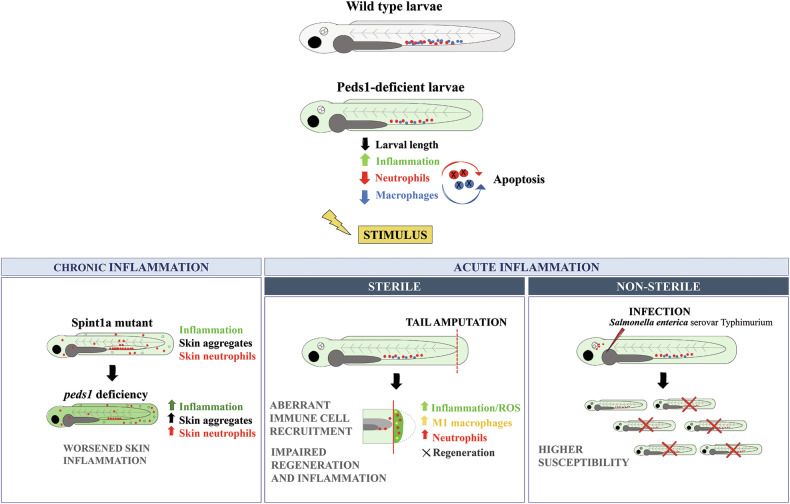
Model showing the effect of inhibiting plasmalogen synthesis under basal and different inflammatory conditions. Inhibition of plasmalogen synthesis promotes a proinflammatory stage with increased neutrophil and macrophage apoptosis. Plasmalogen deficiency also exacerbates chronic skin inflammation, impairs inflammation resolution and tissue regeneration, and results in increased susceptibility to infection.

The role of plasmalogens in inflammation remains controversial, largely because until recently most studies could not distinguish the individual contributions of plasmalogens from those of their alkyl ether lipids precursors. The present study reveals the relevance of endogenous plasmalogens in vertebrate myeloid cell biology and inflammation (Fig. 7). Peds1 deficiency resulted in exacerbated inflammation and delayed development in zebrafish larvae, without provoking apparent morphological alterations. While HSPC emergence and erythropoiesis proceeded normally in Peds1-deficient larvae, they suffered from robust neutropenia and monocytopenia that was rescued by pharmacological inhibition of caspase-3. Specific expression of *peds1b* in neutrophils restored the number of neutrophils, but not of macrophages, in Peds1-deficient larvae, suggesting a cell autonomous effect of Peds1 in myeloid cells and the non-transferability of plasmalogens from neutrophils to macrophages. It has been previously reported that bone marrow-specific deletion of either fatty acid synthase (FAS), which catalyzes the first committed step in de novo lipogenesis, or of peroxisomal reductase activating PPARγ (PexRAP), which acts in ether lipid synthesis following generation of the ether bond by AGPS at the peroxisome, result in neutropenia, but not monocytopenia, due to cell-autonomous loss of neutrophils via endoplasmic reticulum stress and apoptosis [25]. However, since neutropenia was not observed in other mouse models of ether lipid deficiency, such as GNPAT knockout mice, or in patients suffering from RCDP, it was argued that accumulation of the intermediate 1-O-alkyl-glycerone phosphate or tamoxifen toxicity might be responsible for neutrophil death observed in PexRAP-deficient mice [26]. Our results unequivocally show that endogenous plasmalogen biosynthesis is required to maintain neutrophil and macrophage viability in zebrafish larvae.

Importantly, our study reveals that Peds1 deficiency in zebrafish exacerbates both acute and chronic inflammation (Fig. 7), hinting that the diminished plasmalogen levels observed in chronic inflammation-linked diseases might be a cause rather than a consequence of these diseases. Moreover, exogenous addition of plasmalogens not only rescued the exacerbated inflammation of Peds1-deficient larvae, but also reduced inflammation in wild type animals, thus providing additional support for the anti-inflammatory role exerted by PRT in several animal disease models and patient-derived cells studies, and in AD and PD patients [7, 20]. Our results may also have clinical relevance in PRT, since plasmalogens, but not their precursors, mediate the anti-inflammatory effects in zebrafish, as revealed by the failure of the precursors to alleviate inflammation in Peds1-deficient larvae. Our finding that all the plasmalogens used, regardless of the number of double bonds in their acyl chain at *sn*-2, similarly ameliorated skin inflammation, rules out that the observed effects were related to their PUFA content.

The acute inflammation model revealed a critical role of endogenous plasmalogens in myeloid cell activation. Although Peds1-deficient larvae were neutropenic and monocytopenic, early neutrophil and macrophage recruitment to wound was normal. However, inflammation resolution was impaired, as evidenced by neutrophil and Tnfa^+^ macrophage persistence in the wound, and dramatic increased and lasting levels of H_2_O_2_, Il1b and Nfkb in the wound area (Fig. 7). Strikingly, we observed impaired tissue regeneration in Peds1-deficient larvae, which was fully rescued by Il1b deficiency. The role played by Il1b in tail regeneration is complex. While a transient production of Il1b by epithelial cells appears to be required for proper regeneration through the induction of genes encoding important regenerative factors, exacerbated production of Il1b promotes the apoptosis of regenerative cells [27, 28]. In addition, macrophages are required for the attenuation of Il1b production in the wound, thus preventing apoptosis of regenerative cells [27]. Furthermore, the reduced number of recruited macrophages at later stages of regeneration and their skew towards M1-like phenotype may further impair the regeneration process [29]. Whatever the outcome, our results point to a critical role for plasmalogens in tissue regeneration by promoting the resolution of inflammation rather than by a direct effect on cell proliferation and differentiation.

Our results also revealed the importance of plasmalogens in the response to bacterial infection (Fig. 7). Hence, Peds1-deficient larvae were more susceptible to bacterial infection but, more importantly, exogenous plasmalogens not only alleviated this higher susceptibility of Peds1-deficient larvae, but also increased the resistance of wild-type larvae. These effects were again mediated by plasmalogens, since their precursor increased bacterial resistance of wild-type but not of Peds1-deficient larvae. Furthermore, the higher susceptibility of Peds1-deficient larvae to bacterial infection was fully rescued by pharmacological inhibition of caspase-3 and partially rescued by Csf3a-induced myelopoiesis. These results further suggest that plasmalogens are required for neutrophil survival rather than migration, activation and microbicidal functions. The role of plasmalogens in human myeloid cell biology is largely unknown. It has been reported that neutrophil activation results in changes in plasma membrane phospholipids, including phosphatidylethalonamine plasmalogen content [30]. Similarly, the plasmalogen profile was also altered during the differentiation of monocytes to macrophages [31]. In addition, human neutrophils include a unique plasmenylethalonamine pool that undergoes myeloperoxidase-dependent oxidation during activation and is elevated in the plasma during sepsis [32].

In summary, we report for the first time a cell autonomous role of plasmalogens in the survival of vertebrate neutrophils and macrophages, which seems to be dispensable for their recruitment and activation. In addition, while endogenous plasmalogen production was found to be critical to regulate inflammation and promote its resolution, exogenous plasmalogen supplementation exerted beneficial effects by dampening physiological and pathological inflammation and increasing bacterial infection clearance. The zebrafish model developed here is a unique tool to shed light into the relevance of plasmalogens in physiology and disease.

## Methods

### Animals and ethical statement

Zebrafish (*Danio rerio* H.) were obtained from the Zebrafish International Resource Center (ZIR) and mated, staged, raised and processed according to the zebrafish handbook [33]. The lines *Tg(mpx:eGFP)*^*i114*^ [34], *Tg(mpx:Gal4.VP16)*^*i222*^ [35], *Tg(lyz:DsRED2)*^*nz50*^ [36], *Tg(mfap4.1:Tomato)*^*xt12*^ [37], *Tg(gata1a:DsRed)*^*sd2*^ and *Tg(-6.0itga2b:eGFP)*^*la2*^ [38], *Tg(NFkB-RE:eGFP)*^*sh235*^ [39], *Tg(il1b:GFP-F)*^*ump3*^ [40], *Tg(tnfa:eGFP-F)*^*ump5*^ [23], *mitfa*^*w2/w2*^*; mpv17*^*a9/a9*^, referred to here as casper, [41], and *spint1a*^*hi2217Tg/hi2217Tg*^ [21] were previously described. The experiments performed comply with the Guidelines of the European Union Council (Directive 2010/63/EU) and the Spanish RD 53/2013. The experiments and procedures performed were approved by the Bioethical Committees of the University of Murcia (approval number #669/2020).

### Gain- and loss-of-function experiments

Crispr RNA (crRNA) for zebrafish genes *peds1a and peds1b*, *il1b* and negative control **(**Supplementary Table 1**)**, and tracrRNA were purchased from Integrated DNA Technologies (IDT) and resuspended in nuclease-free duplex buffer (DB) up to 100 μM. For duplexing 1 μl of each was mixed and incubated for 5 min at 95 °C. After cooling down to room temperature (RT), the duplex was diluted to 1000 ng/µl by adding 1.43 µl of Nuclease-Free Duplex Buffer. Finally, the injection mix was prepared by mixing 1 μl of duplex, 2.55 μl of DB, 0.25 μl Cas9 nuclease V3 (IDT, #1081058) and 0.25 μl of phenol red, giving final concentrations of 250 ng/μl of gRNA duplex and 500 ng/μl of Cas9. The prepared mix was microinjected into the yolk of one- to eight-cell-stage embryos using a microinjector (Narishige) (0.5–1 nl per embryo). For checking the efficiency of crRNA, genomic DNA from a pool of 10 microinjected larvae was extracted with the HotSHOT method [42] and used as template to amplify the target sequences with a specific set of primers **(**Supplementary Table 1**)**. Sanger sequencing data were analyzed with the TIDE webtoolM (https://tide.nki.nl/) and/or SYNTHEGO Crisper Performance Analysis webtool (https://ice.synthego.com). A double knockout (DKO) zebrafish line was successfully generated using the CRISPR-Cas9 approach. Two crRNAs targeting each *peds1* gene were designed and used to completely delete these genes (Supplementary Table 1). Carriers harboring deletions in both *peds1* genes were identified through genotyping and subsequently crossed to produce a DKO line.

In vitro-transcribed RNA (*csf3a*) was obtained following manufacturer’s instructions (mMESSAGE mMACHINE kit, Ambion) from a *csf3a* construct described previously [43]. RNA was mixed in microinjection buffer (×0.5 Tango buffer and 0.05% phenol red solution) and microinjected into the yolk sac of one-cell-stage embryos using a microinjector (Narishige; 0.5–1 nl per embryo). The same amount of RNA was used in all experimental groups.

The *uas:peds1b; mylf7:eGFP* construct was generated by MultiSite Gateway assemblies using LR Clonase II Plus (ThermoFisher Scientific) according to standard protocols and using Tol2 kit vectors described previously [44]. The *uas:peds1b; mylf7:eGFP* construct (40 ng/µl) was microinjected (0.5–1 nl) into the yolk sac of one-cell-stage embryos together with Tol2 RNA (155 ng/μl) in microinjection buffer. Embryos were sorted at 2 dpf under a fluorescence stereomicroscope using the heart fluorescent marker of the construct.

### Lipid extraction and GC-MS analyses

Each sample of ~50 larvae was collected by centrifugation, washed once with cold PBS and stored at −80 °C until further use. Lipid extraction and fatty acid methyl ester (FAME) analysis were performed following a previously described protocol with slight changes [6]. In brief, the pellets were transferred to Pyrex glass tubes, resuspended in 4 ml of chloroform:methanol (2:1 v/v) by vortexing and incubated for 1 h in ice. Then, they were mixed with 1 ml of 120 mM KCl, vortexed for 10 s and centrifuged at 400 g for 5 min at 4 °C to induce phase separation. After removing the upper aqueous/protein phase, the lower phase was passed through a Whatman No. 1 filter paper into a new Pyrex glass tube and dried under a N_2_ stream at 35 °C. For FAMEs generation, dried samples were subjected to acid hydrolysis at 55 °C overnight in 0.5 ml toluene and 1 ml of 1% H_2_SO_4_ in methanol. The reaction was quenched with 1 ml of 0.2 M KHCO_3_, mixed gently with 2.5 ml hexane:diethyl ether (1:1 v/v) containing 0.01% 2,6-di-tert-butyl-4-methylphenol (BHT; Scharlab) to minimize oxidation and centrifuged at 500 g for 2 min at 4 °C. The upper hexane:ether phase was transferred to a separate tube and the lower phase was re-extracted with 2.5 ml hexane:diethylether (1:1 v/v). The combined upper phase was dried with N_2_ at 35 °C and resuspended in 200 µl hexane. Alternatively, to also analyse ether lipid-derived OAGs, dried samples were resuspended in 162.5 µl hexane and 37.5 µl N-methyl-N- trimethylsilyltrifluoroacetamide (MSTFA) (Sigma-Aldrich) and incubated at 37 °C for 1 h. GC-MS and subsequent data analyses were performed as indicated previously [6]. The determination was performed in 3 biological replicates of 50 pooled larvae.

### Analysis of gene expression

Total RNA was extracted from the tail part of the zebrafish body with TRIzol reagent (Invitrogen) following the manufacturer’s instructions and treated with DNase I, amplification grade (1 U/mg RNA: Invitrogen). SuperScript IV RNase H Reverse Transcriptase (Invitrogen) was used to synthesize first-strand cDNA with random primer from 1 mg of total RNA at 50 °C for 50 min. Real-time PCR was performed with an ABIPRISM 7500 instrument (Applied Biosystems) using SYBR Green PCR Core Reagents (Applied Biosystems). Reaction mixtures were incubated for 10 min at 95 °C, followed by 40 cycles of 15 s at 95 °C, 1 min at 60 °C, and finally 15 s at 95 °C, 1 min 60 °C, and 15 s at 95 °C. For each mRNA, gene expression was normalized to the ribosomal protein S11 (*rps11*) content in each sample using the Pfaffl method [45]. The primers used are shown in Supplementary Table 1. In all cases, each PCR was performed with triplicate samples and repeated with three independent biological replicates.

### Chemical treatments

In some experiments, 24-hours post fertilization (hpf) embryos manually dechorionated were treated with caspase-3 inhibitor (Z-DEVD-FMK, C3I, 50 µM, MedChemExpress), plasmalogens or ether lipid precursor (20 µM, Avanti lipids) from 1–5 days by bath immersion at 28 °C. Incubation was carried out in 6-well plates containing 20 – 25 larvae/well in egg water supplemented with 1% dimethyl sulfoxide (DMSO) as vehicle. Plasmalogens and the custom-synthesized ether lipid precursor were dried under a N_2_ stream at 35 °C and resuspended in DMSO: HsVEPE1 (1-(1*Z*-octadecenyl)-2-oleoyl-*sn*- glycero-3-phosphoethanolamine or 18(Plasm)-18:1 PE), HsVEPE2 (1-(1*Z*-octadecenyl)-2- arachidonoyl-*sn*-glycero-3-phosphoethanolamine or 18(Plasm)-20:4 PE), HsVEPE3 (1-(1*Z*- octadecenyl)-2-docosahexaenoyl-*sn*-glycero-3-phosphoethanolamine or 18(Plasm)-22:6 PE), HsAEPE1 (1-*O*-octadecanyl-2-oleoyl-*sn*-glycero-3- phosphoethanolamine or *O*-18:0-18:1 PE).

### Neutrophil and macrophage staining

Sudan black stains zebrafish neutrophil granules and the procedure was performed as originally reported [46]. Briefly, zebrafish 3 dpf larvae were anesthetized in 0.16 mg/ml buffered tricaine and fixed for 2 h at room temperature in 4% methanol-free formaldehyde. All the larvae were rinsed with PBS three times, incubated for 15 min with Sudan black and washed extensively in 70% EtOH in water. This was followed by progressive rehydration: 50% EtOH in PBS and 0.1% Tween 20 (PBT), 25% EtOH in PBT and PBT alone, and stored at 4 °C until neutrophil counting using a Leica MZ16F fluorescence stereo microscope.

### Neutral red staining

Neutral red stains zebrafish macrophages granules and the procedure was performed as originally reported [47]. Briefly, macrophages staining was performed on live 3 dpf larvae by incubating the larvae in 0.5 mg/ml of neutral red in embryo medium at 25–30 °C in the dark for 5–8 h. The larvae were anesthetized in 0.16 mg ml-1 tricaine and imaged using a Leica MZ16F fluorescence stereo microscope.

### In vivo imaging of zebrafish larvae

To study the total number, the distribution and recruitment of immune cells, 2 or 3 days post fertilization (dpf) transgenic larvae were anesthetized in embryonic medium with 0.16 mg/ml tricaine. Images were taken of the areas of interest (otic region and tail) or of the whole body at different time points, as appropriate considering the study model, using a Leica MZ16F fluorescence stereomicroscope. The number of fluorescent/stained cells was determined by visual counting, and the fluorescence intensity of different parameters (Il1b, Nfkb, and H_2_O_2_) was obtained and analyzed from a region of interest (ROI) with ImageJ (FIJI) software [48]. The number of larvae analyzed are indicated in the figures.

### Tail transection and regeneration assay

For tail transection experiments, 3 dpf larvae anesthetized by tricaine bath immersion were transected from the tail with a sterile micro-scalpel as previously described [49]. At least three independent experiments were performed with a total number of 20–50 larvae per treatment. In vivo neutrophil and macrophage recruitment was evaluated by assessing the recruitment of cells at wound sites (the region posterior to the circulatory loop) at various time points throughout inflammatory process. Macrophages polarization was analyzed by the doble positive fluorescent signal using *Tg(mfap4.1:Tomato)* and *Tg(tnfa:eGFP)* transgenic lines. Tissue regeneration was also evaluated by assessing the regenerated tail fin area at 3, 6, 24 and 48 h post-wounding (hpw) using Image J software. Regeneration was calculated by analyzing the regenerated tail fin area vs. fin areas of unamputated animal.

### Oxidative stress assay

H_2_O_2_ release at the wound site was quantified employing the live cell fluorogenic substrate acetyl-pentafluor-obenzene sulphonyl fluorescein (Cayman Chemical), as previously described [50]. Briefly, larvae of 3 dpf were incubated with 50 µM of the substrate reagent prior tail fin amputation procedure for 1 h in a 24-well plate, then immediately injured. To determine oxidative response post injury at the wound intensity of fluorescence was assessed using Image J software.

### Infection assay

For the infection experiments, *Salmonella enterica* serovar Typhimurium strain 12023 (wild type, WT) was used. Overnight cultures in Luria-Bertani (LB) broth were diluted 1/5 in LB with 0.3 M NaCl, incubated at 37 °C until 1.5 optical density at 600 nm was reached, and finally diluted in sterile PBS. Larvae of 2 dpf were anaesthetized in embryo medium with 0.16 mg ml−1 tricaine and 10 bacteria (yolk sac, systemic infection) or 200 (otic vesicle, myeloid cell recruitment) per larvae were microinjected. Larvae were allowed to recover in egg water at 28–29 °C and monitored for clinical signs of disease or mortality over 5 days. Neutrophil and macrophage recruitment to the otic vesicle was analyzed as described above for tail wounding. At least three independent experiments were performed with a total number of 50 larvae per treatment.

### Statistical analysis

Statistical analysis was performed using Prism 8.0 (GraphPad Software, CA, USA). No calculation was performed to predetermine sample size; experiments were repeated three times to ensure robustness. Embryos were randomly allocated to each experimental condition and analyzed in blinded samples. No data were excluded from the analysis. Data are shown as mean ± s.e.m. and were analyzed by analysis of variance and a Tukey multiple range test to determine differences between groups. The differences between two samples were analyzed by the two-sided Student’s *t*-test. The data met normal distribution assumption when required and showed similar variances. A log-rank test was used to calculate the statistical differences in the survival of the different experimental groups. A *p*-value of <0.05 was considered statistically significant.

### Supplementary information

Figures S1-S3

Table S1

## Data Availability

The datasets generated and/or analyzed during the current study are available from the corresponding author (VM) on reasonable request.
