# Supplementary material for: Peds1 deficiency in zebrafish results in myeloid cell apoptosis and exacerbated inflammation
Source: Cell Death Discov. 2024 Aug 29;10:388. doi: 10.1038/s41420-024-02141-w (PMC11362147; doi:10.1038/s41420-024-02141-w)
Supplement: Supplementary file 2 — Table S1 [file 41420_2024_2141_MOESM2_ESM.pdf]

Supplementary Table 1. crRNAs and primers used in this study. The gene symbols followed the Zebrafish Nomenclature Guidelines (http://zfin.org/zf_info/nomen.html).

Gene	ENA or Ensembl accesion number	Name	Sequence (5'→3')	Model	Use
peds1a	ENSDARG00000011498	Dr.Cas9.SI:CH211-212O1.2.1.AQ	TCCCCAATGGACCATCCCAG	Crispants	Generation of crispants/ KO lines
peds1b	ENSDARG00000042732	Dr.Cas9.TMEM189.1.AC	CCAGGTGGAAGTATGTTACC	Crispants	
peds1a	ENSDARG00000011498	CD.Cas9.QVPC3716.AA	AATTCTTAGATAAGAGTAGC	KO	
peds1a	ENSDARG00000011498	CD.Cas9.BGBB3586.AA	AGCAATTTATGTAAGGCGGT	KO	
peds1b	ENSDARG00000042732	CD.Cas9.QTMF9623.AA	CGTGTTGTGCTATCTTGACC	KO	
peds1b	ENSDARG00000042732	CD.Cas9.VWWP0161.AA	CCACATTAAGATCTGACTCC	KO	
il1b	ENSDARG00000098700	Dr.Cas9.IL1B.1.AA	CAGGCCGTCACACTGAGAGC	Crispants	
peds1a	ENSDARG00000011498	F	TGTGCCTCTCACTCTTCATTGT	Crispants	Genetic edition efficiency
		R	ACTGTCGACTAAGCAAAATCTCC	Crispants	
peds1b	ENSDARG00000042732	F	CACACAGTGAGACCAGCTAATCTTT	Crispants	
		R	CACCATACCGCCCAGGTCTA	Crispants	
peds1a	ENSDARG00000011498	F	GGCGTTTGTGTCTTATCAGCG	KO	
		R	TCACTTGAAGGAACCCCAGA	KO	
peds1b	ENSDARG00000042732	F	GTCACTCATTGCAATCTACCAGC	KO	
		R	GAGGACAGAGTTCAGTAAGCAG	KO	
il1b	ENSDARG00000098700	F	CATGATGACTTTTGTGGAGAGAAAA	Crispants	
		R	GTAACCTGTACCTGGCCTGC	Crispants	
rps11	NM_213377.1	F	ACAGAAATGCCCCTTCACTG	Crispants/ KO lines	RT-qPCR
		R	GCCTCTTCTCAAAACGGTTG		
il1b	NM_212844.2	F	GCCTGTGTGTTTGGGAATCT		
		R	TGATAAACCAACCGGGACA		
nfkb1	ENSDARG00000105261.2	F	TTCTTCTTGGTCACGTGCAG		
		R	ACTCTCAGCATCCGCATCTT		
tnfa	NM_212859.2	F	GCGCTTTTCTGAATCCTACG		
		R	TGCCCAGTCTGTCTCCTTCT		
cxcl8a	XM_001342570.7	F	GTCGCTGCATTGAAACAGAA		
		R	CTTAACCCATGGAGCAGAGG		
